# TRPV1 Downregulation Impairs Prostate Cancer Growth: Functional and Translational Insights from Cellular and *In Vivo* Models

**DOI:** 10.7150/ijbs.125429

**Published:** 2026-01-22

**Authors:** Belén G. Sánchez, José M. Mora-Rodríguez, Alicia Bort, Ana Palacín, Carlos Sánchez-Rodríguez, Manuel Sánchez-Chapado, Julie Courraud, Jerome Zoidakis, Inés Díaz-Laviada

**Affiliations:** 1 University of Alcalá, School of Medicine and Health Sciences, Department of Systems Biology, Biochemistry and Molecular Biology Unit, 28871 Alcalá de Henares, Madrid, Spain. Health Research Institute of Castilla-La Mancha (IDISCAM), Spain.; 2 Príncipe de Asturias Hospital, Department of Urology, Alcalá de Henares, Madrid, Spain.; 3 University of Alcalá, School of Medicine and Health Sciences, Department of Surgery, Medical and Social Sciences, 28871 Alcalá de Henares, Madrid, Spain.; 4 Section of Clinical Therapeutics, Department of Medicine, National and Kapodistrian University of Athens, Alexandra hospital, Athens, Greece.; 5 Proteomics Core Facility, School of Science, National and Kapodistrian University of Athens, Greece.; 6 Section of Biochemistry and Molecular Biology, Department of Biology, National and Kapodistrian University of Athens, Greece.; 7 Proteomics Laboratory, Biomedical Research Foundation, Academy of Athens, Greece.

**Keywords:** TRPV1, biomarker, capsaicin, prostate cancer, proliferation, cell cycle, stemness, proteomics

## Abstract

The transient receptor potential vanilloid 1 (TRPV1), the canonical capsaicin (CAP) receptor, has been implicated across diverse pathologies, yet its role in prostate cancer (PCa) remains elusive. Here, we uncover TRPV1 as a key regulator of PCa progression and a mediator of CAP's antiproliferative effects. Through a comprehensive strategy combining proteomic profiling, Transgenic Adenocarcinoma of the Mouse Prostate (TRAMP) mouse modeling, and validation in human prostate biopsies, we assessed TRPV1 expression, its functional role, and its association with tumor markers. Both proteomic analysis and Western blotting of TRPV1-silenced cells revealed reduced expression of PCNA, Cyclin B1, and AURKA, along with elevated levels of the cell cycle inhibitor p21. Similarly, CAP treatment resulted in comparable changes in the proteomic profile. Functional assays demonstrated that both TRPV1 knockdown and CAP exposure significantly impaired cell cycle progression and mitosis. Moreover, sustained CAP treatment led to a reduction in TRPV1 expression, further supporting its oncogenic role. In TRAMP mice, a high-fat diet feeding elevated plasma PSA levels and TRPV1 expression in the prostate, whereas CAP supplementation reversed these effects. Importantly, TRPV1 expression correlated positively with cancer stem cell markers in both murine models and human samples. Collectively, our results reveal that TRPV1 is not only overexpressed in PCa but also contributes to proliferation regulation and stemness features, positioning it as a potential diagnostic and prognostic biomarker for prostate cancer.

## Introduction

Prostate cancer (PCa) is the second leading cause of cancer-related death in men, with incidence rates increasing by 2-3% annually. Approximately 300,000 new PCa cases are estimated for 2025 in the United States 1. Conventional therapies include chemical castration and surgical resection; however, these therapies inevitably fail in a large proportion of patients. In about 30% of cases, an aggressive, recurrent, castration-resistant form of PCa develops that is resistant to antiandrogen therapy and represents a major therapeutic challenge. Despite notable advancements in the treatment and management of PCa, most tumors still progress, resulting in approximately 375,000 deaths worldwide annually 2-4. Therefore, new treatments strategies are needed to manage this devastating disease.

Owing to their low toxicity and ability to inhibit multiple oncogenic pathways, natural phytochemicals have emerged as promising therapeutics agents 5, 6. Capsaicin (8-methyl-N-vanillyl-trans-6-nonenamide), the pungent compound present in hot chili peppers, has been shown to exert antitumor effects across various cancer types, including PCa 7, 8. Notably, capsaicin exhibits superior efficacy in inhibiting the growth of PCa cells compared to other natural compounds 9. Capsaicin induces autophagy blockage and apoptosis in prostate cancer PC3 cells, inhibits the growth of castration-resistant PCa cells 10, 11, and facilitates the degradation of the androgen receptor 12. In addition, it sensitizes human PCa cells to both radiotherapy 13 and chemotherapy 14, 15. However, it may play a dual role in tumorigenesis, acting either as a carcinogen or as a cancer-preventive agent 8.

Capsaicin binds to the transient receptor potential vanilloid 1 (TRPV1) cation channel, functioning as a chemical agonist and inducing the entrance of calcium into the cell 16, thereby activating many intracellular pathways. Interestingly, capsaicin can also operate independently of TRPV1 by regulating membrane fluidity, ion flux, and intracellular levels of reactive oxygen species 17. For example, capsaicin and its analogs have been shown to alter antioxidant capacity in prostate cells, increase catalase activity, and markedly reduce the activity of NADPH-generating enzymes 18. Despite these findings, the role of TRPV1 in mediating the antitumor effects of capsaicin remains unclear.

TRPV1 is a ligand-activated membrane cation channel that was cloned by David Julius in 1997 19, a discovery for which he was awarded the Nobel Prize in Physiology and Medicine in 2021. TRPV1 assembles as a homotetramer, with each subunit comprising six transmembrane (TM) domains and a pore-forming loop between TM5 and TM6, as well as intracellular N- and C-terminal regions 20. TRPV1 responds to a variety of stimuli, including noxious heat (>43 °C), low extracellular pH (pH ≤ 5.9), and numerous endogenous and exogenous ligands (vanilloids, proinflammatory lipid mediators, endocannabinoids, and plant and animal toxins) 21. Capsaicin binds intracellularly to a hydrophobic cavity in TRPV1 formed by the TM3 and TM4 domains 20-22, eliciting a burning pain sensation 23. Prolonged or repeated activation of the TRPV1 leads to its desensitization. TRPV1 opening is modulated by several processes, such as phosphorylation and binding of ATP, calcium-calmodulin and phosphoinositides 24. Phosphoinositide lipids interact with the vanilloid binding site, acting as positive cofactors for TRPV1 25. Calcium influx triggers phospholipase C activation, depleting the agonists phosphatidylinositol 4,5-bisphosphate and phosphatidylinositol 4-phosphate, thus restricting channel function and causing desensitization 26, 27. High concentrations of capsaicin result in sustained TRPV1 desensitization in a calcium-dependent manner due to repeated channel activation 28.

Although TRPV1 is primarily expressed in sensory neurons responsible for detecting noxious stimuli, it is also present in non-neuronal cells such as T lymphocytes, epithelial cells, endothelial cells, muscle cells, pancreatic cells, adipocytes, and spermatozoa 16. In the normal prostate, TRPV1 expression is low 29, but elevated levels have been reported in PCa tissues 30, 31 and derived cell lines 30, 32. Furthermore, higher TRPV1 expression has been associated with poorer prognosis in PCa patients 32. These findings suggest a role for TRPV1 in regulating PCa cell growth and proliferation.

Nonetheless, the precise interplay between TRPV1 and the antiproliferative effects of capsaicin remains unclear. In this study, using a proteomics-based approach, we explore TRPV1-associated pathways that may underlie its prognostic and therapeutic relevance. Our results indicate that TRPV1 functionally contributes to PCa cell proliferation by modulating pathways related to DNA replication and mitosis. Moreover, positive correlations between TRPV1 expression and markers of stemness and drug resistance support its potential as a prognostic biomarker and therapeutic target in PCa.

## Materials and Methods

### Materials

Capsaicin (CAP) was purchased from Sigma-Aldrich (St. Louis, MO, USA). The psPAX2 vector was a gift from Didier Trono (Addgene, Watertown, MA, USA, plasmid #12260; http://n2t.net/addgene:12260, accessed on 24 January 2022; RRID:Addgene_12260), pCMV-VSV-G was a gift from Robert Weinberg 33 (Addgene plasmid #8454; http://n2t.net/addgene:8454, accessed on 24 January 2022; RRID:Addgene_8454) and the pLKO.1-TRC cloning vector was a gift from David Root 34 (Addgene plasmid #10878; http://n2t.net/addgene:10878, accessed on 24 January 2022; RRID:Addgene_10878). The TRPV1_OHu19934D_pcDNA3.1+/C-(K)-DYK plasmid used for TRPV1 overexpression was obtained from GenScript (Piscataway, NJ, USA). All solvents and acids used for mass-spectrometry were of LC-MS grade and were obtained from Sigma-Aldrich, similar to the proteomics-related reagents. Trypsin Gold was purchased from Promega (Madison, WI, USA).

### Cell cultures

The PNT2 human prostate epithelial cell line was obtained from the European Collection of Authenticated Cell Cultures (ECACC, Salisbury, UK). The human prostate cancer cell lines LNCaP (CRL-1740), PC3 (CRL-1435), and DU-145 (HTB-81) were purchased from the American Type Culture Collection (ATCC, Manassas, VA, USA). LN-CSS cells were generated by culturing LNCaP cells in RPMI-1640 supplemented with 10% charcoal-stripped fetal bovine serum (FBS) for more than three months. LN-FLU cells were obtained by culturing LNCaP cells in RPMI-1640 supplemented with 10% FBS and 2 μM 2-hydroxyflutamide for nine months.

All cell lines were routinely cultured in RPMI-1640 medium supplemented with 100 IU/mL penicillin G sodium, 100 µg/mL streptomycin sulfate, 0.25 µg/mL amphotericin B (Invitrogen, Waltham, MA, USA) and 10% FBS (Sigma-Aldrich, St. Louis, MO, USA) at 37 °C in 5% CO_2_. *Mycoplasma* contamination was routinely assessed. For the treatment experiments, cells were seeded and allowed to adhere for 24 h, after which the medium was replaced with serum-free RPMI-1640 and treated with 80 μM CAP for 1, 12, 24 or 48 h, depending on the specific experimental design.

### Western blot analysis

For Western blotting, proteins were extracted by lysing cells in buffer (50 mM Tris pH 7.4, 0.8 M NaCl, 5 mM MgCl_2_, 0.1% Triton X-100) supplemented with protease and phosphatase inhibitor cocktails (Roche Diagnostics, Mannheim, Germany). Lysates were incubated on ice for 15 minutes and clarified by microcentrifugation. Protein concentration was determined using the Bradford Protein Assay Kit (Bio-Rad, Hercules, CA, USA). Twenty micrograms of total protein were resolved by SDS-polyacrylamide gel electrophoresis (SDS-PAGE) and subsequently transferred onto a PVDF membrane. Next, membranes were incubated overnight at 4 °C with primary antibodies (**Table 1**). After washing with T-TBS, membranes were incubated with peroxidase-conjugated anti-mouse or anti-rabbit secondary antibodies (1:5000) for 2 h at room temperature (**Table 1**). Detection of the immune complexes was performed via the Clarity^TM^ Western ECL Substrate (Bio-Rad) and imaged with the ChemiDoc MP Imaging System (Bio-Rad). Band intensities were quantified using ImageJ software (National Institutes of Health, Bethesda, MD, USA) and expressed as fold change relative to control conditions.

### RNA extraction and reverse transcription quantitative polymerase chain reaction

Total RNA was extracted using the NZY Total RNA Isolation Kit (NZYtech, Lisbon, Portugal) following the manufacturer's instructions. Two micrograms of total RNA were reverse-transcribed into cDNA using the NZY First-Strand cDNA Synthesis Kit (NZYtech). Quantitative PCR (RT-qPCR) was conducted in a 10 μL reaction volume using NZY Speedy qPCR Green Master Mix (2x) (NZYtech) on a 7500 Real-Time PCR System (Applied Biosystems Inc., Foster City, CA, USA) according to the manufacturer's instructions. Primer sequences used for amplification are listed in **Table 2**.

### Immunocytochemistry

Cells were fixed in 4% paraformaldehyde in PBS and permeabilized with 0.1% Triton X-100. Immunolabeling with a TRPV1 antibody (dilution 1:250, Thermo Fisher Scientific, Waltham, MA, USA) was conducted through incubation at room temperature for 1 hour. Then, samples were incubated with an Alexa Fluor 488-conjugated secondary antibody (dilution 1:500) and DAPI (dilution 1:2000, Invitrogen). Subsequently, the coverslips were mounted with Mowiol mounting medium (Sigma-Aldrich). Imaging procedures were carried out with a Leica TCS SP5 laser-scanning confocal microscope with LAS-AF imaging software utilizing a 40× oil immersion objective.

For lysosome labeling, cells were incubated with 100 nM LysoTracker (Life Technologies, Thermo Fisher Scientific). To label the endoplasmic reticulum, cells were treated with CellLight ER-RFP BacMam 2.0 reagent (Invitrogen) at a concentration of 40 particles per cell per 1 × 10⁸ cells and incubated for 16 hours. After organelle labeling, immunocytochemistry protocol was carried out as described above.

### Lentivirus transduction

A lentiviral transduction system was employed to generate cell lines with TRPV1 silencing. Lentiviral particles were produced in HEK293T cells by cotransfecting the plasmids of interest with helper plasmids. To produce lentiviruses for TRPV1 silencing, the following transfection mixture was prepared and added to a 10 cm dish of HEK293T cells at approximately 70% confluence: 5 μg of psPAX2, 3 μg of pCMV-VSV-G, and 10 μg of either the empty pLKO.1-TRC cloning vector or the pLKO.1-TRC cloning vector containing shTRPV1. The shTRPV1 sequence was designed based on clone ID: TRCN0000044190 (Sigma-Aldrich). Polyethylenimine (PEI) (Polysciences, Warrington, PA, USA) at 1 mg/mL was used at a 3:1 ratio relative to the total DNA in the mixture. Six hours post-transfection, the medium was replaced with fresh culture medium. Viral supernatants were collected at 48 and 72 hours post-transfection, filtered through a 0.45-µm pore membranes, and used to infect target cells. Polybrene (1 μg/mL, Sigma-Aldrich) was added during infection to increase transduction efficiency. Following infection, cells were expanded to larger culture surfaces and selected with puromycin (3 μg/mL, STEMCELL Technologies, Vancouver, BC, Canada) 24 hours later.

### Proteomic analysis

Two independent proteomic comparisons were performed (5-6 biological replicates). First, we compared the proteomic profiles of PC3 prostate cancer cells transduced with the empty vector (EV) versus PC3 cells transduced with the vector encoding shRNA targeting TRPV1 (shTRPV1). Second, we analyzed differential protein expression in PC3 cells treated with 80 µM CAP for 24 h versus vehicle-treated controls (DMSO).

PC3 cells were seeded at 1 × 10⁶ cells per 10-cm dish, rinsed with PBS, detached using trypsin, and centrifuged at 300 × g for 5 minutes. The supernatant was discarded, and cell pellets were frozen at -80°C. The cell pellets were lysed with trifluoroacetic acid (TFA, ≥99%) (Thermo Fisher Scientific) and intensely vortexed until complete dissociation. Subsequently, lysates were neutralized by adding 8 volumes of 2 M Tris base, and protein concentrations were determined using the Lowry assay 35. A total of 4 μg of protein per sample was transferred to a 96-well plate for direct proteomics and diluted to 50 μL with 0.1 M Tris base. Proteins were reduced with 10 mM tris-2(-carboxyethyl)-phosphine (TCEP), alkylated with 40 mM chloroacetamide (CAA), and digested with trypsin overnight at 37°C (10 ng trypsin per μg protein). Peptides were purified using C18 stage tips prepared by packing five C18 discs into 200-μL pipette tips. Stage tips were conditioned sequentially with 250 μL of acetonitrile, 250 μL of solvent B (40% acetonitrile, 0.5% acetic acid, 60% water) and 250 μL of solvent A (0.5% acetic acid in Milli-Q water), followed by centrifugation at 2700 × g for 5 minutes at each step. Peptides obtained after digestion were acidified to pH 2-3 with TFA, loaded onto the C18 stage tips, and centrifuged at 2700 × g. Columns were then washed with 250 μL of solvent A. Peptides were eluted with 40 μL of solvent B, dried under vacuum, and stored at -20°C. Prior to nanoLC-MS/MS analysis, the peptides were reconstituted in 40 μL of solvent A. A total of 500 ng of purified peptides was injected onto a 25-cm reverse-phase C18 column (nanoElute 2, Bruker Daltonics) and separated over a 30-minute gradient, as described previously 36.

Data acquisition was performed on a timsTOF fleX (Bruker Daltonics) via library-free data-independent acquisition (DIA) with parallel accumulation serial fragmentation (PASEF). Proteins identification was performed with FragPipe v22.0 37, 38 using the UniProt-reviewed human reference proteome (UP000005640, downloaded on Dec. 02, 2024). The exact parameters are described elsewhere 36. Protein quantification was conducted via DIA-NN v1.9 39, applying a stringent false discovery rate (FDR < 1%) at both the peptide and protein levels. As performed by default by FragPipe, peptide levels were normalized via the MaxLFQ 40 algorithm.

Statistical and bioinformatic analyses of the proteomic data were performed using the FragPipe-Analyst platform (https://fragpipe-analyst.org/) and the R programming environment (version 4.4.2). Proteins were filtered to ensure a minimum of 50% of non-missing values in at least one group (five/six technical replicates per group). The abundances were further normalized via the variance stabilizing normalization method. The remaining missing values were imputed via the Perseus-like method.

Subsequent comparative analyses between experimental groups, as performed via FragPipe-Analyst, included fold-change calculations, ANOVA, pairwise moderated t tests with adjustment for multiple comparisons (FDR method), principal component analysis, and Pearson correlation.

For downstream analyses in R, custom scripts were adapted from publicly available templates. The following R packages were employed: tidyverse, pheatmap, clusterProfiler 41, 42, BiocManager and enrichplot 43. These packages were used to identify differentially expressed proteins (DEPs), generate volcano plots and heatmaps, and conduct functional enrichment analyses of Gene Ontology (GO) terms and Kyoto Encyclopedia of Genes and Genomes (KEGG) pathways. All R scripts, analysis templates, and raw/processe data files are available in the GitHub repository linked to this study: https://github.com/Belen-G-Sanchez/https-github.com-Proteomics_Analysis.git, ensuring full transparency, reproducibility, and traceability of the findings.

### Cell viability

Cell proliferation was evaluated using the MTT assay. Briefly, cells were seeded at a density of 1.5 × 10^5^ cells per well in 12-well plates. At the specified time points post-seeding, 100 μL of MTT solution (3-(4,5-dimethyl-2-thiazolyl)-2,5-diphenyl-2H-tetrazolium bromide; Sigma-Aldrich) was added to each well. Plates were incubated at 37°C for 1 h to allow for formazan crystal formation. After incubation, the medium was removed, and the resulting formazan crystals were solubilized in 2-propanol. The optical density of each well was measured at a wavelength of 595 nm using a microplate reader (iMark, Bio-Rad). Cell viability was expressed as a percentage relative to control cells.

### Transient transfection of TRPV1

LNCaP cells were transfected with 2.5 μg of a TRPV1 plasmid using 5 μL Lipofectamine 3000 (Invitrogen). Cells were harvested 48 h post-transfection for Western blotting or RT-qPCR. An anti-FLAG antibody (F3165; working dilution 1:2500) was purchased from Sigma-Aldrich.

### Animal experiment design

Male TRAMP (transgenic adenocarcinoma of the mouse prostate) mice were obtained from The Jackson Laboratory at 5-8 weeks of age (C57BL/6-Tg(TRAMP)8247Ng/J strain 003135). Animals were housed in groups of three per cage in a laminar airflow cabinet, maintained on a 12-hour light/dark cycle at 21-23°C and 40-60% humidity, with *ad libitum* access to food and water.

One week after arrival, mice were divided into 4 experimental groups based on the diet to be administered. The diets were obtained from the Central Research Services (University of Almería, Spain) (**Table 3**). The base composition of all the diets was the AIN-93M diet formula for adult mice. For the CAP-supplemented diet, capsaicin was added at a concentration of 0.01% w/w (**Table 3A**). The high-fat diet was formulated by reducing carbohydrate content to 52% through a decrease in corn starch and increasing the lipid content to 24% by adding pork fat (**Table 3B**). The four experimental groups were as follows: standard diet (STD), STD supplemented with 0.01% w/w capsaicin (STD + CAP), high-fat diet with 24% lipids (HFD), and HFD supplemented with 0.01% w/w capsaicin (HFD + CAP). At 8 weeks of age, mice were switched from the STD to their respective experimental diets. Body weight and uneaten food weight were recorded weekly to ensure that daily food intake was similar across all groups.

Following six months of dietary intervention, animals were euthanized by CO₂ inhalation in a dedicated chamber. Prostate glands were then excised and processed for Western blotting and RT-qPCR analyses.

All experimental procedures were approved by the Ethics Committee of the University of Alcalá and the Ethics Committee of the Community of Madrid (PROEX 131.8/23). Animal handling and experimentation adhered to Spanish regulations (RD) for the housing, care, and use of laboratory animals, in full compliance with European Community guidelines. The UK Coordinating Committee on Cancer Research guidelines were also strictly followed. Animal welfare was assessed daily using a panel of 10 indicators. In cases of adverse effects, pain, or distress (score of 15 out of 40), humane endpoints were applied.

### Determination of PSA levels in mouse plasma

Plasma levels of prostate-specific antigen (PSA) were measured using a Mouse PSA ELISA Kit (Elabscience®, Houston, TX, USA; catalogue number: E-EL-M0961), following the manufacturer's instructions. Samples were assayed in duplicate. Absorbance was measured at 450 nm using an iMark™ microplate reader (Bio-Rad). Standard curves and concentrations were determined via Microplate Manager® 6 software, version 6.3 (Bio-Rad).

### Human prostate sample collection

A total of 17 human prostate samples were obtained from patients undergoing radical prostatectomy at the Hospital Universitario Príncipe de Asturias (HUPA), Alcalá de Henares, Spain. Following surgical resection, tumor staging was determined by a pathologist using the Gleason scoring system, and tumor specimens were collected from the region with the highest tumor burden. Non-tumoral prostate tissues were sampled from areas distant from the tumor site within the same prostate. All tissue samples were immediately stored at -80°C until further analysis. The study was conducted in accordance with the principles of the Declaration of Helsinki and approved by the Ethics Committee of HUPA (PROCARE v.2.0.), as well as by the Research and Animal Experimentation Committee of the University of Alcalá (CEIP/2024/6/126).

### Statistical analysis

All the statistical analyses, except those related to the proteomic data (detailed in the Proteomic Analysis section), were performed via GraphPad Prism version 8.0 (GraphPad Software, La Jolla, CA, USA). Statistical comparisons between two groups were performed using the unpaired Student's t test. Comparisons involving three or more groups were conducted using one-way or two-way ANOVA, followed by Sidak's or Tukey's multiple comparisons test or appropriate *post hoc* tests. A p-value < 0.05 was considered statistically significant. Statistically significance is indicated in figures as follows: *p* < 0.05 (*), *p* < 0.01 (**), *p* < 0.001 (***), *p* < 0.0001 (****). For clarity, only the most relevant pairwise comparisons are displayed in the figures.

## Results

### TRPV1 expression is elevated in prostate cancer cells

**Figure 1:**
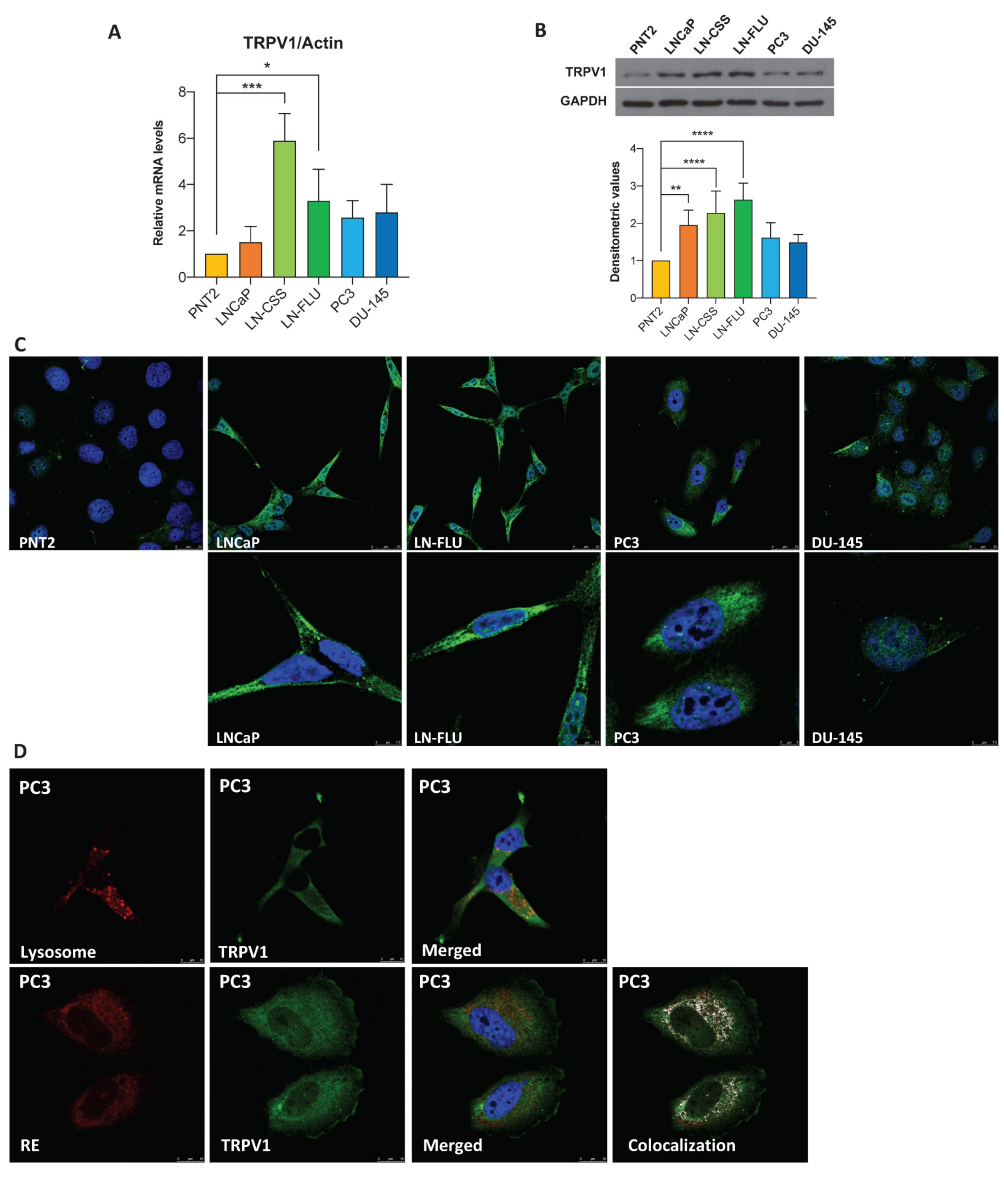
** TRPV1 expression in prostate cell lines. (A)** TRPV1 expression levels were determined by RT-qPCR. Data represent relative expression normalized to actin, used as the housekeeping gene. Results are presented as mean ± SD of three independent experiments. **(B)** TRPV1 protein expression was analyzed by Western blotting, with GAPDH as a loading control. The healthy prostate cell line PNT2 was used for comparison. A representative blot from three independent experiments is shown. **(C)** Fluorescence microscopy images showing TRPV1 labeling (green) in PNT2, LNCaP, LN-FLU, PC3, and DU-145 cells. Nuclei were stained with DAPI (blue). **(D)** Top row: lysosomes labeled with LysoTracker (red) and nuclei stained with DAPI (blue). Bottom row: PC3 cells transfected with CellLight ER-RFP BacMam to visualize the endoplasmic reticulum. A representative image from two independent experiments is shown. *p* < 0.05 (*), *p* < 0.01 (**), *p* < 0.001 (***) and *p* < 0.0001 (****) indicate significant differences via one-way ANOVA.

To assess TRPV1 expression in prostate cancer (PCa), we analyzed six cell lines: one non-tumorigenic prostate line (PNT2) and five tumorigenic lines representing various stages of PCa progression. LNCaP cells correspond to an early-stage, androgen-sensitive model of prostate cancer, despite originating from a lymph node metastasis. LN-CSS and LN-FLU cells, derived from LNCaP, have acquired resistance to androgen deprivation therapy (ADT) 44 and exhibit neuroendocrine-like features, representing a model of therapy-induced phenotypic plasticity in prostate cancer. PC3 and DU-145 cells represent the most aggressive stages of the disease, originating from bone and brain metastases, respectively. We evaluated TRPV1 expression by RT-qPCR, Western blotting, and immunocytochemistry. RT-qPCR (**Figure 1A**), Western blotting (**Figure 1B**), and immunocytochemistry (**Figure 1C**) revealed increased TRPV1 expression levels in cancerous cells compared with noncancerous cells, with the highest TRPV1 expression observed in ADT-resistant LN-CSS and LN-FLU cells.

Although TRPV1 has traditionally been considered a plasma membrane protein, increasing evidence indicates its expression is not confined to the cell surface 45. Consistent with this, **Figure 1C** shows strong intracellular staining in prostate cells. To determine whether this intracellular TRPV1 localizes to specific organelles, we performed double staining: red for cellular structures (lysosomes or endoplasmic reticulum (ER)) and green for TRPV1 (**Figure 1D**). This assay was conducted in PC3 cells, as they proliferate in a monolayer, facilitating protein labeling by immunocytochemistry. Lysosomal staining did not overlap with intracellular TRPV1, whereas a strong co-localization was observed with the ER. These results indicate that, in addition to the plasma membrane, TRPV1 is present in the ER of prostate cancer cells, suggesting potential intracellular roles that merit further functional investigation.

### TRPV1 modulation induces changes in the proteomic profile of PC3 cells

To further investigate the pathways in which TRPV1 is involved in PCa pathogenesis, we performed a proteomic analysis of PC3 cells infected with either an empty vector (EV) or a shTRPV1 vector (shTRPV1) designed to reduce TRPV1 expression (**Supplementary Figure 1**). A total of approximately 5,430 proteins, corresponding to 50,181 precursor peptides, were identified and quantified under stringent confidence thresholds (FDR < 1% and 99% confidence).

Principal component analysis (PCA) (**Supplementary Figure 2A**) revealed a clear segregation between EV and shTRPV1 groups, with the first principal component (PC1) accounting for 73.3% of the variance and distinguishing the samples primarily by the type of vector used. Intra-group variability was minimal (PC2: 10.5%, non-imputed data), and correlation matrix analysis (**Supplementary Figure 2B**) confirmed the tight clustering of biological replicates within each condition. Despite overall high correlation between groups, TRPV1 silencing induced significant changes in the proteomic profile, as demonstrated by hierarchical clustering analysis of the two groups.

**Figure 2:**
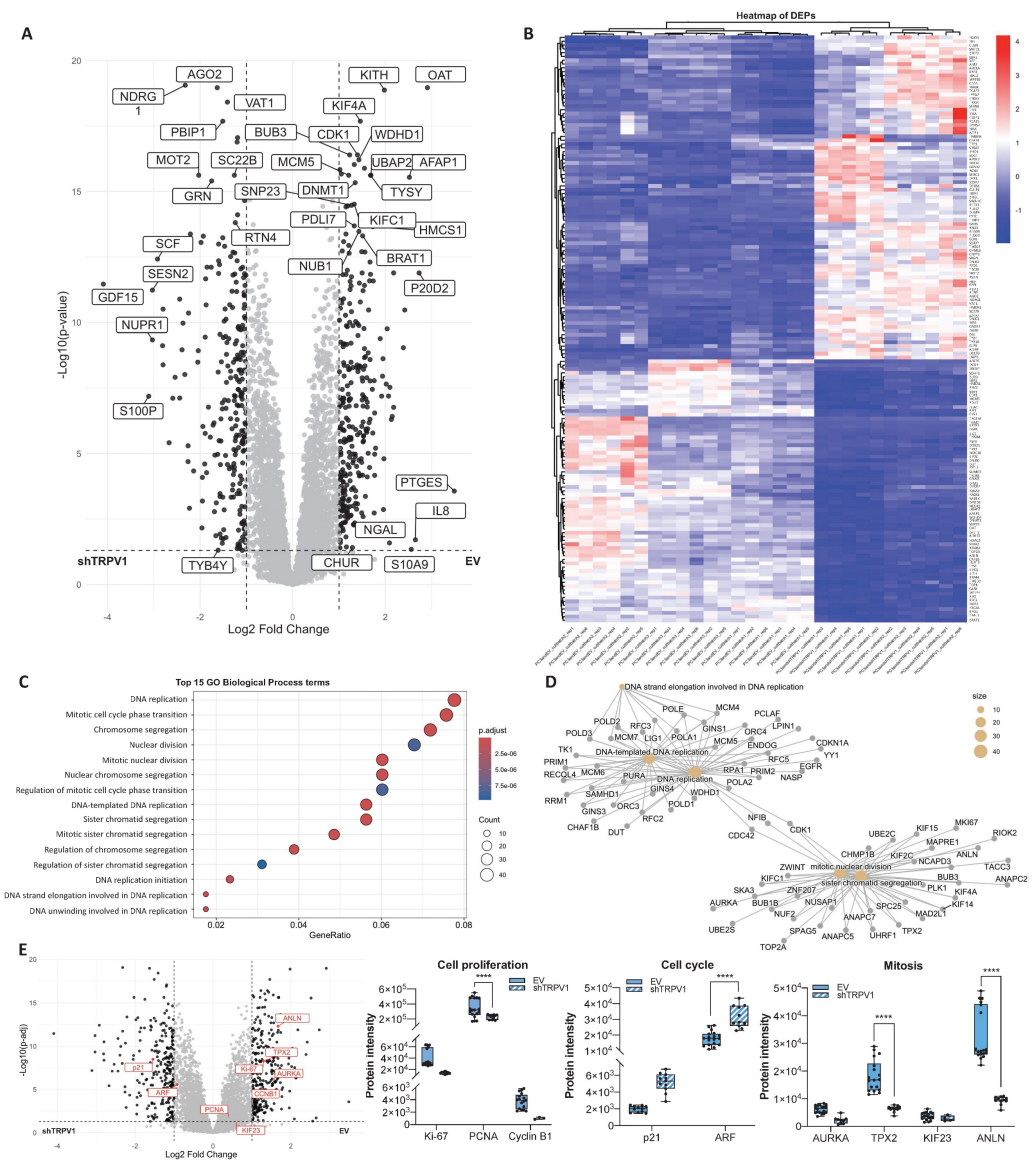
** Proteomic analysis of PC3 cells infected with the empty vector (EV) or with the vector containing shTRPV1 (shTRPV1). (A)** Volcano plot comparing protein expression between PC3 cells infected with the EV or shTRPV1 vector. Differentially expressed proteins (DEPs) with a log2 fold change > |1| and *p* < 0.05 are shown in black. **(B)** Heatmap of DEPs with a log2-fold change > |1.2|, showing hierarchical clustering of samples and proteins. **(C)** Gene Ontology (GO) enrichment analysis of biological processes associated with the DEPs between EV- and shTRPV1-infected cells. The top 15 enriched GO terms are shown. Dot size indicates the number of DEPs involved in each term, while color intensity reflects statistical significance (adjusted p-value). **(D)** Network diagram illustrating the biological processes associated with DEPs. Yellow nodes represent enriched biological processes, and the other nodes represent individual DEPs. **(E)** Left: Volcano plot highlighting in red proteins involved in cell proliferation (Ki-67, PCNA, and Cyclin B1), cell cycle regulation (p21 and ARF), and mitosis (AURKA, TPX2, KIF23, and ANLN). DEPs with a log2- fold change > |1| and *p* < 0.05 are shown in black. Right: Box plots representing protein intensity levels of the red highlighted proteins. EV-transduced cells are represented in solid blue, whereas shTRPV1-transduced cells are shown in striped blue. *p* < 0.05 (*), *p* < 0.01 (**), *p* < 0.001 (***) and *p* < 0.0001 (****) indicate significant differences via two-way ANOVA and Sidak's multiple comparisons test. Six biological replicates per condition (EV or shTRPV1) from two independent experiments were analyzed.

To identify differentially expressed proteins (DEPs) between EV and shTRPV1 cells, a log2 fold change threshold of |1| and adjusted *p* < 0.05 was applied. The volcano plot (**Figure 2A**) illustrates the distribution of 543 identified DEPs, of which 294 were more highly expressed in EV cells and 249 were upregulated in shTRPV1 cells. The heatmap (**Figure 2B**, zoom from Figure 2B in **Supplementary Figure 3**) shows hierarchical clustering of the samples into two distinct groups, “EV” and “shTRPV1”, with well-defined expression profiles.

To identify the biological processes associated with the DEPs, we performed GO enrichment analysis. As shown in **Figure 2C**, DEPs between the EV and shTRPV1 groups were related primarily to DNA replication and mitosis. The enriched terms were further analyzed via a network diagram (**Figure 2D**), which revealed that the most significantly altered processes between the two conditions were “*DNA-templated DNA replication*”, “*DNA replication*”, “*mitotic nuclear division*” and “*sister chromatid segregation*”. Complementary KEGG pathway enrichment analysis (**Supplementary Figure 2C**) corroborated these findings, revealing significant modulation of pathways related to the cell cycle, DNA replication and multiple DNA damage repair processes (including “*nucleotide excision repair*”, “*mismatch repair*” and “*base excision repair*”).

To specifically evaluate whether TRPV1 is involved in proliferation- and cell cycle-related processes, we analyzed several key proteins associated with these functions in greater detail. Box plots of protein intensities (**Figure 2E**) revealed a significant decrease in the proliferation marker Proliferating Cell Nuclear Antigen (PCNA) in shTRPV1-infected cells, together with a significant increase in the cell cycle inhibitor ARF. These alterations were accompanied by significantly reduced levels of proteins essential for mitosis and proper mitotic spindle formation, such as TPX2 and Anillin (ANLN), in cells with low TRPV1 expression.

### Knockdown of TRPV1 halts PCa cell proliferation

**Figure 3:**
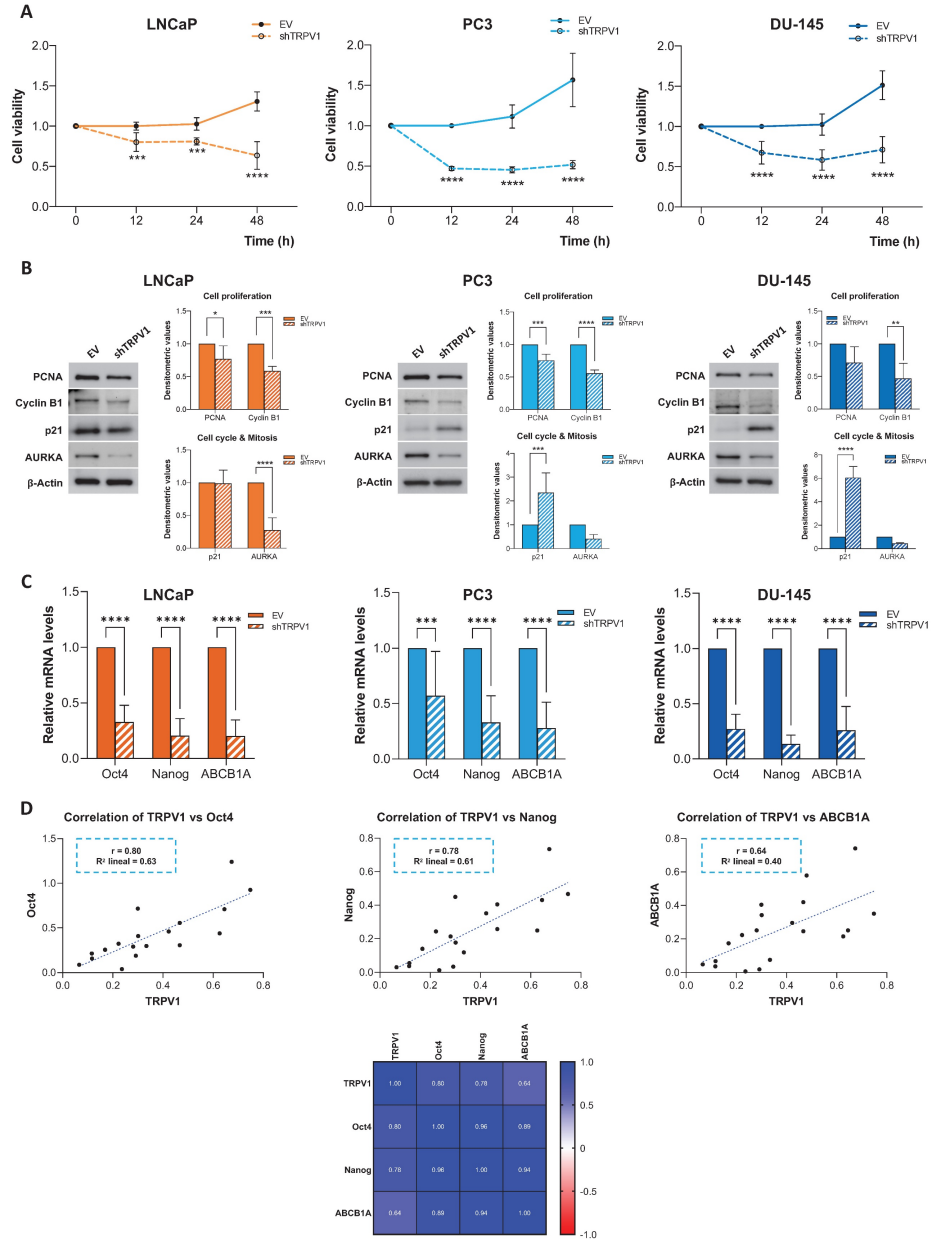
** Effect of TRPV1 receptor silencing on cell viability and proliferation.** LNCaP, PC3, and DU-145 cells were infected with either an empty vector (EV) or a shTRPV1 vector via lentivirus-mediated transduction. **(A)** Cell viability of prostate cancer cells. Following infection, the cells were seeded at equal densities, and MTT assays were performed at 12, 24, and 48 hours post-seeding to evaluate the impact of TRPV1 silencing on cell proliferation. Data from non-silenced and silenced cells were normalized to 1 to facilitate the comparison. The mean ± SD of three independent experiments is shown. **(B)** Protein expression levels of proteins involved in proliferation, cell cycle regulation and mitosis. The levels of the proteins were determined by Western blotting, and β-actin served as a loading control. The densitometric analyses of the bands represent the mean ± SD of three different experiments. **(C)** Relative mRNA expression levels of the stemness markers Oct4, Nanog and ABCB1A. mRNA levels were quantified via RT-qPCR, normalized to actin (housekeeping gene) and presented as mean ± SD of six independent experiments. **(D)** Correlation analysis between TRPV1 and the stemness markers Oct4, Nanog and ABCB1A in TRPV1-silenced cells. Top: Scatter plots showing Pearson's correlation coefficients (r) for TRPV1 vs. Oct4, TRPV1 vs. Nanog and TRPV1 vs. ABCB1A. Bottom: Heatmap displaying Pearson's correlation coefficients for TRPV1, Oct4, Nanog and ABCB1A expression. *p* < 0.05 (*), *p* < 0.01 (**), *p* < 0.001 (***) and *p* < 0.0001 (****) indicate significant differences according to two-way ANOVA and Sidak's multiple comparisons test.

To validate the role of TRPV1 in cell viability suggested by the proteomic data, we silenced TRPV1 expression in LNCaP, PC3, and DU-145 cells (**Supplementary Figure 1**) and assessed cell viability at different time points (12, 24, and 48 h) (**Figure 3A**). The MTT assay results revealed that TRPV1 knockdown significantly reduced the proliferation of LNCaP, PC3, and DU-145 cells as early as 12 hours. This effect persisted over time, and by 48 hours, the reduction in cell proliferation was more pronounced.

The effects of TRPV1 silencing on proliferation, cell cycle progression, and mitosis were further confirmed by Western blot analysis of key regulatory proteins (**Figure 3B**). TRPV1 downregulation led to a significant decrease in the proliferation markers PCNA and cyclin B1 across all the cell lines, with the decrease in PCNA expression being marginally significant in DU-145 cells. The cell cycle inhibitor p21 was significantly upregulated in TRPV1-silenced PC3 and DU-145 cells, suggesting potential cell cycle arrest; however, its expression remained unchanged in LNCaP cells. Finally, we observed a reduction in the expression of Aurora kinase A (AURKA) in all TRPV1-knockdown cell lines, with a significant decrease in LNCaP cells. These findings confirmed the results obtained from the proteomic analysis and pointed to a relevant role of TRPV1 in PCa cell proliferation.

To investigate whether TRPV1 expression in prostate cancer cells is associated with stemness-related features, the mRNA levels of the pluripotency regulators Oct4 and Nanog, as well as ABCB1A, a key mediator of resistance to anticancer treatments, were analyzed. As shown in **Figure 3C**, TRPV1 silencing led to a reduction in the relative mRNA levels of Oct4, Nanog, and ABCB1A. Given the similar expression patterns observed for TRPV1 and these genes, correlation analyses were performed (**Figure 3D**). TRPV1 expression showed positive correlations with Oct4 (Pearson correlation coefficient r = 0.80), Nanog (r = 0.78), and ABCB1A (r = 0.64).

To further confirm the relationship between TRPV1 and markers of stemness and resistance to antitumor treatments, the opposite approach was undertaken. LNCaP cells were transiently transfected with a plasmid overexpressing TRPV1. Transfection efficiency was confirmed by detection of the FLAG tag by Western blotting (**Supplementary Figure 4A**), and the mRNA levels of Oct4, Nanog, and ABCB1A were quantified by RT-qPCR. Cells overexpressing TRPV1, and therefore expressing FLAG, exhibited a significant increase in the expression of these markers (**Supplementary Figure 4B**).

### Capsaicin modifies the proteomic profile of PCa cells

One of the agonists of the TRPV1 receptor is the natural compound capsaicin (CAP), which we previously described as having antitumor activity in PCa cells 14, 46. To investigate the pathways and biological processes affected by CAP treatment, we performed a proteomic analysis of PC3 cells treated with 80 μM CAP or with vehicle (DMSO) for 24 hours. A total of approximately 5,164 proteins corresponding to 51,604 precursor peptides were identified, all of which were validated under stringent thresholds (FDR < 1% and 99% confidence).

The PCA plot (**Supplementary Figure 5A**) revealed that the differences observed between the DMSO and CAP groups were primarily attributed to CAP treatment (PC1: 40.7%, non-imputed data) and the variability between biological replicates within each condition (PC2: 22.9%, non-imputed data). Correlation matrix analysis (**Supplementary Figure 5B**) confirmed that the DMSO-treated cells formed a distinct group from the CAP-treated cells, with replicates being closely clustered in each group.

**Figure 4:**
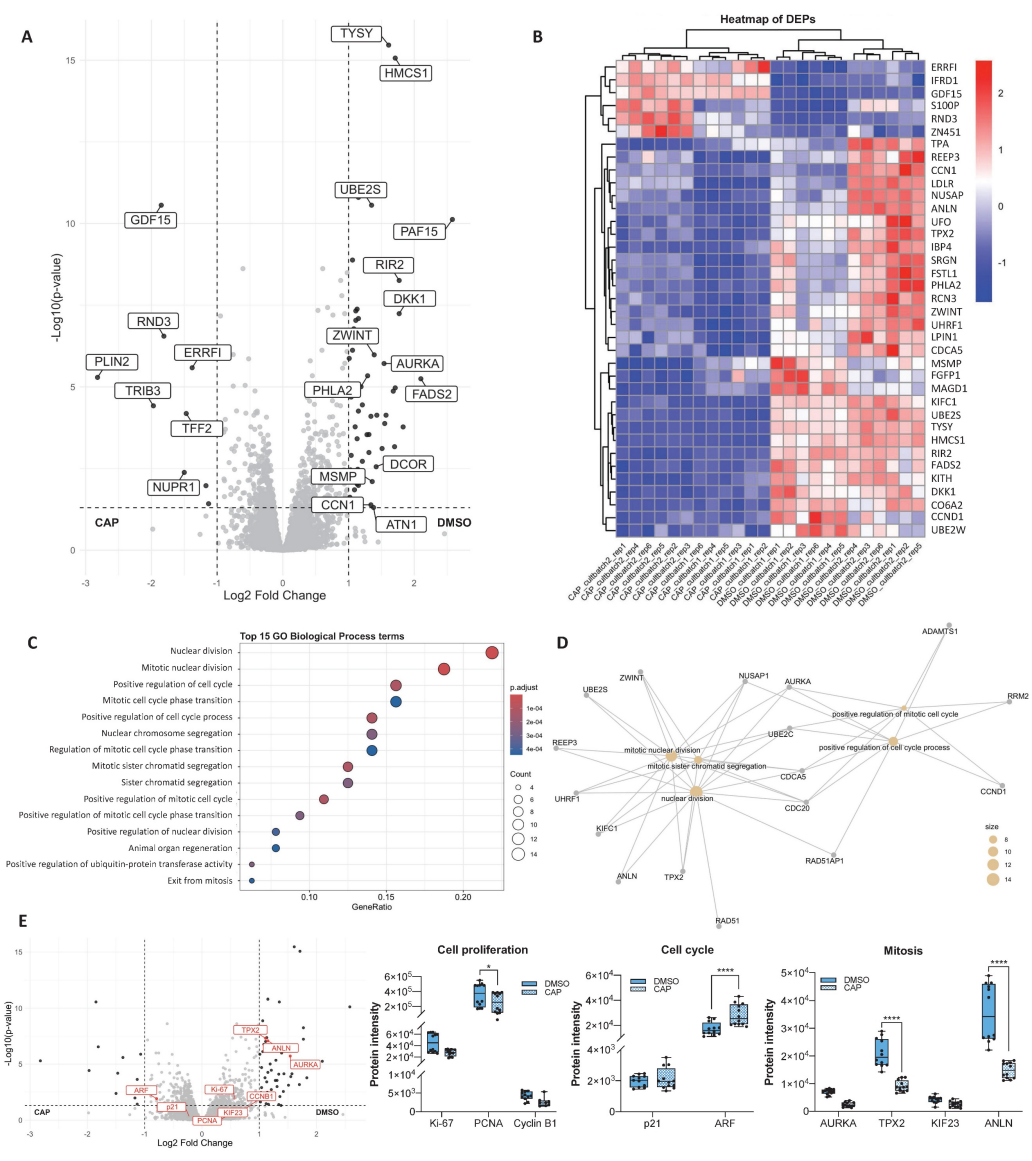
** Proteomic analysis of PC3 cells treated with capsaicin. (A)** Volcano plot comparing protein expression between PC3 cells treated with DMSO (vehicle) or with capsaicin (CAP, 80 µM). Proteins differentially expressed (DEPs) with a log2-fold change > |1| and p < 0.05) are shown in black. **(B)** Heatmap of DEPs (log2-fold change > |1|), showing hierarchical clustering of samples and proteins. **(C)** Gene Ontology (GO) enrichment analysis for biological processes associated with DEPs, displaying the top 15 enriched GO terms. The gene ratio indicates the proportion of DEPs associated with each GO term relative to the total number of DEPs. Larger dots indicate a greater number of associated proteins and color intensity reflects statistical significance (p.adjust). **(D)** Network diagram illustrating the biological processes associated with the DEPs. Yellow nodes represent GO terms (biological processes), whereas the other nodes correspond to the associated DEPs. **(E)** Left: Volcano plot highlighting in red selected proteins involved in cell proliferation (Ki-67, PCNA, and Cyclin B1), cell cycle regulation (p21 and ARF), and mitosis (AURKA, TPX2, KIF23, and ANLN). DEPs with log2-fold change > |1| and p < 0.05 are shown in black. Right: Box plots showing protein intensity levels of the red proteins. DMSO-treated cells are represented in solid blue, whereas CAP-treated cells are shown in striped blue. *p* < 0.05 (*), *p* < 0.01 (**), *p* < 0.001 (***) and *p* < 0.0001 (****) indicate significant differences via two-way ANOVA and Sidak's multiple comparisons test. Six biological replicates per condition (DMSO or CAP) from two independent experiments were analyzed.

The volcano plot (**Figure 4A**) revealed 64 DEPs, fewer than those identified in the comparison between EV-infected and shTRPV1-infected cells. Among these DEPs, 53 proteins were highly expressed in DMSO-treated cells, whereas 11 were upregulated in CAP-treated cells. Although the number of DEPs between the two populations was lower when a log2 fold change threshold of |1| and *p* < 0.05 was used, the heatmap (**Figure 4B**) revealed that the samples still clustered hierarchically into the two treatment groups with clearly differentiated expression profiles.

GO enrichment analysis of biological processes (**Figure 4C**) revealed that DEPs between DMSO- and CAP-treated cells were involved primarily in cell cycle regulation, mitosis, and regulation of protein ubiquitination. Interestingly, several of these processes overlapped with those altered by TRPV1 silencing. Network analysis (**Figure 4D**) highlighted significant alterations in "*nuclear division*", "*mitotic nuclear division*", and "*positive regulation of cell cycle processes*".

To further investigate the effects of CAP on proliferation and cell cycle-related processes, we analyzed the expression of key proteins involved in these pathways (**Figure 4E**). CAP treatment led to a significant reduction in PCNA levels, along with a marked increase in ARF and a decrease in TPX2 and ANLN. Notably, these alterations closely resembled those observed upon TRPV1 silencing.

### Prolonged CAP exposure reduces TRPV1 expression

**Figure 5:**
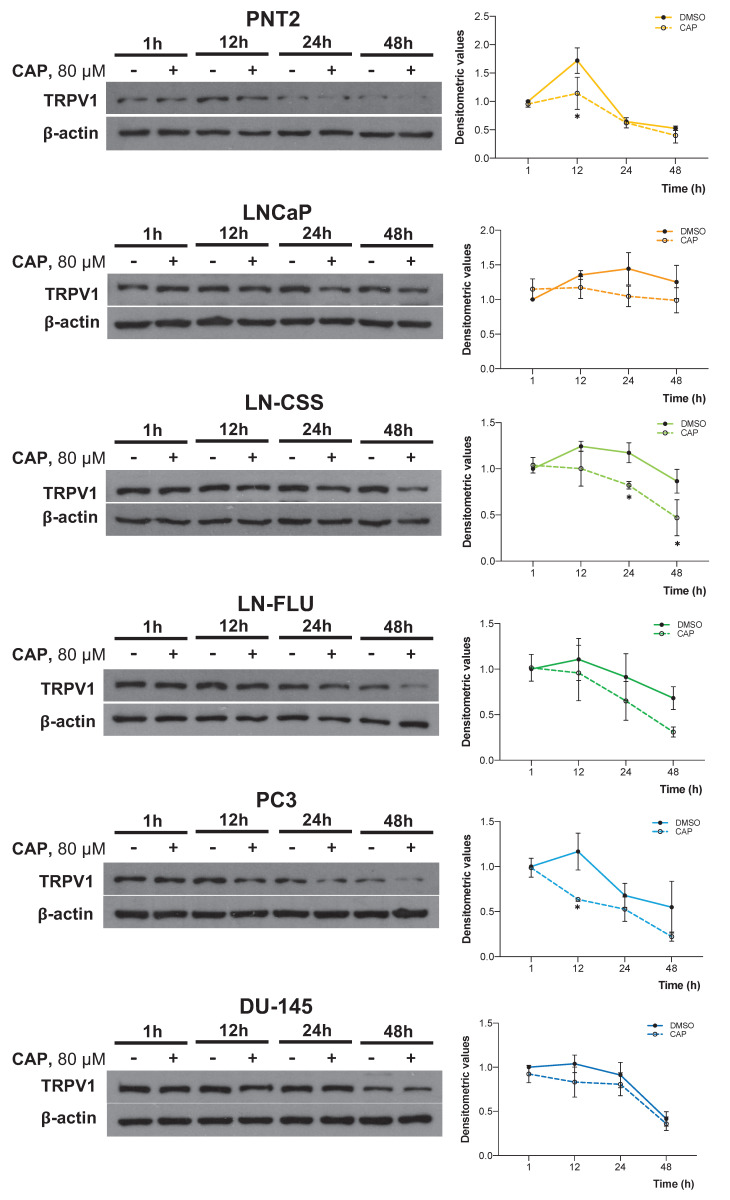
** Time-dependent effect of capsaicin on TRPV1 expression.** Prostate cell lines were treated with 80 µM capsaicin for 1, 12, 24, or 48 hours. TRPV1 protein levels were assessed by Western blot analysis, with actin used as a loading control. A representative blot from three independent experiments is shown. Data are presented as the mean ± SD of three independent experiments. *p* < 0.05 (*), *p* < 0.01 (**), *p* < 0.001 (***) and *p* < 0.0001 (****) indicate significant differences via two-way ANOVA and Sidak's or Tukey's multiple comparisons test.

Given that both TRPV1 silencing and CAP treatment had similar effects on multiple pathways, we investigated whether CAP treatment influenced TRPV1 expression in PCa cells. To this end, cells were treated with 80 μM CAP for 1, 12, 24, or 48 hours, and TRPV1 expression was assessed by Western blotting. As shown in **Figure 5**, CAP treatment progressively reduced TRPV1 expression levels across all PCa cell lines, with a more pronounced effect observed at 24 and 48 hours in LNCaP (*p*_24h_ = 0.1254, *p*_48h_ = 0.5262), LN-CSS (*p*_24h_ = 0.0378, *p*_48h_ = 0.0156), LN-FLU (*p*_24h_ = 0.7055, *p*_48h_ = 0.3182), and PC3 (*p*_24h_ = 0.9493, *p*_48h_ = 0.2325) cells. These results suggest that the antiproliferative effects of CAP may be mediated, at least in part, by sustained TRPV1 downregulation, which becomes more pronounced with prolonged CAP exposure.

### *In vivo* effect of CAP treatment

**Figure 6:**
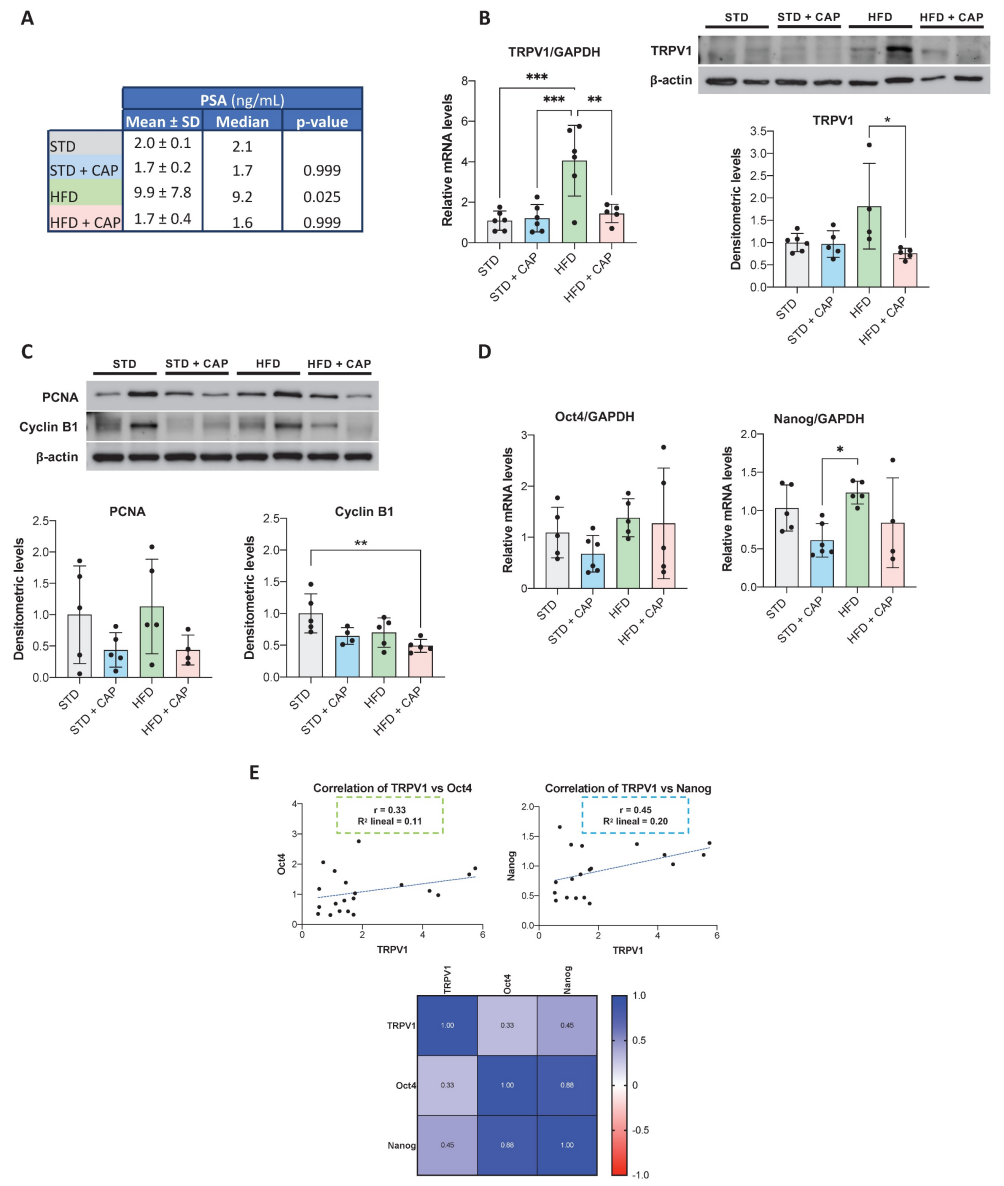
** PSA plasma levels and TRPV1 expression in the prostates of TRAMP mice fed different diets. (A)** PSA levels in plasma measured by ELISA at the end of the experiment. Results are expressed as mean ± SD (n = 6 per group) along with median values. *p*-values comparing each group to the STD group are shown in the rightmost column. **(B)** Left: TRPV1 mRNA levels determined by RT-qPCR, normalized to GAPDH (housekeeping gene) and presented as mean ± SD (n = 6 per group). Right: TRPV1 protein levels assessed by Western blotting with β-actin used as a loading control. A representative blot from two independent samples per group is shown. Densitometric values (mean ± SD) relative to the STD group are presented. **(C)** Protein levels of PCNA and Cyclin B1, key regulators of proliferation and the cell cycle, analyzed via Western blotting. A representative blot from two samples per group is shown with densitometric values (mean ± SD) relative to the STD group. **(D)** Relative mRNA expression levels of the stemness markers Oct4 and Nanog in prostate tissues from TRAMP mice. mRNA levels were quantified via RT-qPCR, normalized to GAPDH (housekeeping gene) and presented as mean ± SD (n = 6 per group). **(E)** Correlation analysis between TRPV1 and the stemness markers Oct4 and Nanog in prostate tissues of TRAMP mice. Top: Scatter plots showing Pearson's correlation coefficients (r) for TRPV1 vs. Oct4 and TRPV1 vs. Nanog. Bottom: Heatmap displaying Pearson's correlation coefficients for TRPV1, Oct4, and Nanog expression. *p* < 0.05 (*), *p* < 0.01 (**), *p* < 0.001 (***) and *p* < 0.0001 (****) indicate significant differences via one-way ANOVA.

The impact of CAP on TRPV1 expression *in vivo* was evaluated in TRAMP mice, a model in which neuroendocrine prostate tumors spontaneously develop from puberty onwards. At 8 weeks of age, mice were assigned to four dietary groups for six months: STD, STD + CAP, HFD, and HFD + CAP. At the end of the experiment, plasma levels of prostate-specific antigen (PSA) were quantified by ELISA to assess PCa incidence across groups. As shown in **Figure 6A**, HFD group presented significantly elevated plasma PSA levels compared with the others groups. However, the HFD + CAP restored the plasma PSA levels back to the control values in the STD group.

TRPV1 receptor expression in the prostate tissue was evaluated by RT-qPCR (**Figure 6B**, left) and western blotting (**Figure 6B**, right). The results revealed a significant increase in TRPV1 mRNA levels in the HFD group relative to STD group. However, HFD + CAP successfully reduced both TRPV1 mRNA and protein expression levels in the prostate compared to HFD group. As shown in **Figure 6C**, the effects of CAP-supplemented diets on key proteins involved in proliferation and the cell cycle were analyzed by Western blotting. The results revealed that PCNA expression in prostate tissue was reduced in the STD + CAP (*p* = 0.4550) and HFD + CAP (*p* = 0.3272) groups compared to STD and HFD, respectively. Cyclin B1 expression was also significantly lower in the HFD + CAP group than in the STD group.

To investigate whether TRPV1 expression in the mouse prostate is related to the acquisition of cancer stem cell characteristics, we quantified the mRNA levels of Oct4 and Nanog (**Figure 6D**). In the left panel of **Figure 6E**, we evaluated the correlation between TRPV1 and Oct4 mRNA levels, whereas in the right panel, we assessed the correlation between TRPV1 and Nanog mRNA levels. The results revealed that TRPV1 was weakly positively correlated with Oct4 and Nanog, with Pearson correlation coefficients (r) of 0.33 and 0.45, respectively. Additionally, as expected, a strong correlation between the two pluripotency markers, Oct4 and Nanog was observed in the heatmap (r = 0.88).

Taken together, these results suggest that TRPV1 expression is upregulated in HFD-fed animals, in which PSA and stem cell markers are also increased, indicating a link between TRPV1 upregulation, more aggressive prostate cancer phenotypes, and a potential theragnostic role for TRPV1 modulation.

### Basic characteristics of the patients

To assess the clinical relevance of our findings in human PCa and their potential translational application, we analyzed TRPV1 expression in tumor and adjacent non-tumor tissues from a cohort of 17 PCa patients. The clinical and pathological characteristics of the patients are summarized in **Table 4**. The mean age was 66.5 years (range, 55 - 75 years), with an average body weight of 84.4 kg (range: 64-112 kg) and a mean body mass index (BMI) of 29.2 kg/m² (range, 23.7-37.9 kg/m²). The Gleason scores, as determined by a pathological examination, ranged from 3+3 to 5+4. Serum PSA levels averaged 12.6 ng/mL, with values ranging from 4.6 to 21.2 ng/mL.

### TRPV1 expression in human prostate cancer biopsies

**Figure 7:**
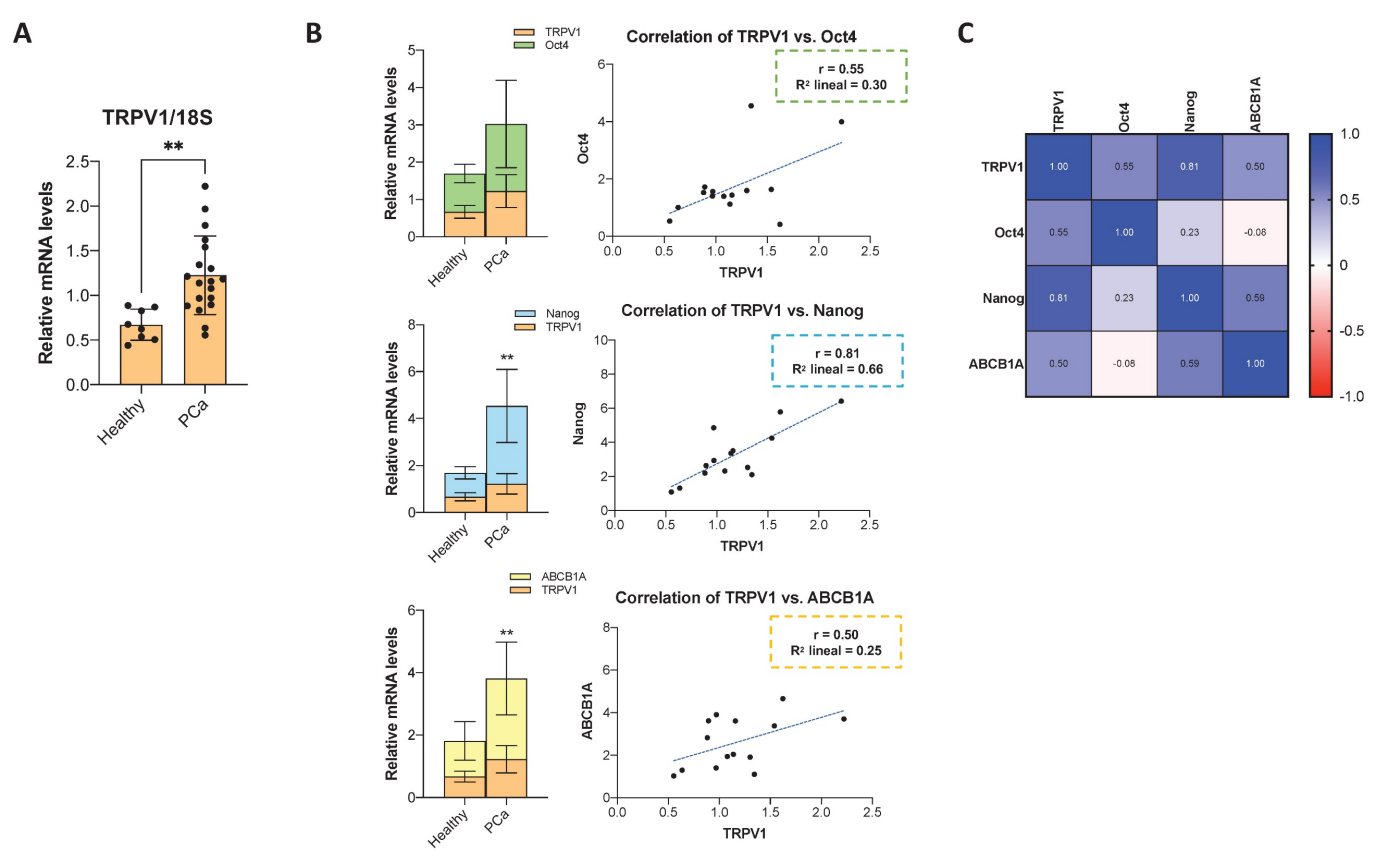
** Expression of TRPV1, cancer stem cell markers, and drug resistance markers in human prostate cancer samples, and their correlations. (A)** Relative TRPV1 mRNA levels in human prostate cancer samples compared to adjacent non-tumor tissues. RT-qPCR data represent relative mRNA expression normalized to 18S as a housekeeping gene. Data are presented as the mean ± SD of 5 healthy tissue samples and 17 different tumor samples. *p* < 0.05 (*), *p* < 0.01 (**), *p* < 0.001 (***) and *p* < 0.0001 (****) indicate significant differences according to the unpaired t test. **(B)** Left: mRNA levels of Oct4, Nanog, and ABCB1A in PCa versus adjacent normal tissues. *p* < 0.05 (*), *p* < 0.01 (**), *p* < 0.001 (***) and *p* < 0.0001 (****) indicate significant differences via two-way ANOVA and Sidak's multiple comparisons test. Right: Scatter plots showing the correlations between TRPV1 and Oct4, Nanog and ABCB1A expression levels in PCa tissues. **(C)** Heatmap showing Pearson's correlation coefficients (*r*) among TRPV1, Oct4, Nanog, and ABCB1A expression in prostate tumor samples (n=17). Correlations were assessed only in tumor tissues.

A fraction of the prostate biopsy sample was dissociated for RNA extraction, followed by RT-qPCR analysis. TRPV1 mRNA levels were significantly elevated in tumor tissues compared to adjacent normal prostate tissue (**Figure 7A**). To further explore the relationship between TRPV1 and cancer stemness, we quantified the mRNA levels of the pluripotency markers Oct4 and Nanog, as well as the multidrug resistance transporter ABCB1A. As shown in **Figure 7B**, Oct4, Nanog, and ABCB1A mRNA levels exhibited a pattern consistent with that of TRPV1, being significantly higher in PCa samples than in normal tissues. Correlation analysis presented as a heatmap (**Figure 7C**) revealed moderate positive correlations between TRPV1 and Oct4 (r = 0.55) and ABCB1A (r = 0.50), with a stronger correlation observed between TRPV1 and Nanog (r = 0.81). These data support a potential role of TRPV1 in PCa tumorigenesis and its association with stemness features.

## Discussion

The TRPV1 receptor is involved in various physiological functions, including pain perception, thermosensation, and energy homeostasis. However, its expression and activity are altered in various diseases, such as epilepsy, atherosclerosis, obesity, insulin resistance, asthma, and cancer 47. Recent studies have demonstrated that the TRPV1 receptor plays a significant role in tumor biology through various pathways 48, 49; however, the molecular mechanisms and specific functions of TRPV1 in different types of cancer remain poorly understood 49. Capsaicin (CAP), a natural TRPV1 agonist with well-documented antitumor properties, exerts effects via both TRPV1-dependent and -independent mechanisms. Additionally, CAP induces TRPV1 desensitization in sensory neurons, leading to analgesic effects. To clarify these apparent discrepancies, we conducted *in vitro* and *in vivo* studies employing state-of-the-art techniques.

TRPV1 mRNA and protein are expressed in many cancer cell lines, and their expression levels differ between healthy and tumor tissues in several types of cancer. These findings suggest that TRPV1 is involved in key processes of cancer progression 49. Some studies have reported that TRPV1 overexpression may have a beneficial effect by downregulating the proliferation of melanoma, intestinal epithelial, and pancreatic cells 50. Nevertheless, increased TRPV1 expression compared with healthy tissue has been observed in lung adenocarcinoma 51, brain tumors, pancreatic cancer and pancreatitis, squamous cell carcinoma of the tongue, and breast cancer 49, 50. In colorectal cancer, TRPV1 gain-of-function promotes tumorigenesis. Moreover, receptor levels are correlated with colorectal cancer progression and may influence patients' clinical prognosis 52. In line with these studies, our data revealed greater expression of TRPV1 in PCa cell lines than in control cells and increased TRPV1 expression in tumor samples from patients. Data from The Human Protein Atlas 53 based on the analysis of 480 PCa samples revealed that low TRPV1 expression was associated with improved patient survival, whereas elevated TRPV1 levels were correlated with poorer prognosis 54. This finding is in accordance with the results of Baker et al., who reported that higher TRPV1 expression was associated with a poor prognosis in PCa patients, suggesting that it could serve as a selective marker for more aggressive cancers 32. These findings support the positive correlation we found in our study between TRPV1 mRNA levels and the pluripotency and stemness markers Oct4 and Nanog, as well as the ABCB1A transporter.

TRPV1 has been reported to be involved in both apoptotic cell death and proliferation. For example, TRPV1 induces apoptosis through mitochondrial dysfunction and membrane depolarization, ER stress, caspase activation, and DNA damage. Conversely, TRPV1 promotes proliferation by activating P2Y2 and EGFR, leading to intracellular protein signaling cascades 55. Our results of the proteomic analysis of PC3 cells infected with shTRPV1 revealed that reducing TRPV1 expression in PCa cells led to a significant decrease in biological processes related to DNA replication and mitosis, supporting the positive effect of TRPV1 on cell growth. Furthermore, we demonstrated that TRPV1 silencing reduces cell viability *in vitro* and halts the proliferation of LNCaP, PC3, and DU-145 cells while decreasing the expression of proliferation and mitosis markers and increasing that of the cell cycle inhibitor p21. These findings are consistent with observations in non-small cell lung cancer, which reported that TRPV1 silencing reduced the viability of H1299 and A549 lung cells and significantly suppressed colony formation, whereas TRPV1 overexpression had the opposite effect 51. Additionally, other studies have shown that the TRPV1 antagonist capsazepine exerts various anticancer effects on multiple tumor cell lines by blocking TRPV1 56.

The TRPV1 agonist CAP is widely known to exert antitumor effects on a wide variety of cancers, including PCa 14. Here, we show that proteomic analysis of CAP-treated cells revealed that CAP also decreases the expression of proteins involved primarily in mitosis, with effects similar to those observed upon TRPV1 silencing. Additionally, CAP induced a time-dependent decrease in TRPV1 expression, which became significant after 24 hours of treatment. These findings suggest that CAP induces a long-term TRPV1 desensitization, decreasing TRPV1 levels and therefore exerting effects similar to those of TRPV1 knockdown. Under normal conditions, membrane-bound TRP channels can undergo endocytosis for degradation by the proteasome and lysosome or be recycled to the cell surface via endosomes. CAP can induce both acute and long-term TRPV1 desensitization. Acute desensitization depends on intracellular calcium, whereas long-term desensitization relies on factors such as TRPV1 endocytosis and degradation 45. The altered regulation of protein ubiquitination observed via proteomic analysis of CAP-treated cells further supports the notion that CAP induces TRPV1 long-term TRPV1 downregulation.

Previous studies have linked a HFD to increased PCa development and progression, as well as reduced survival rates in TRAMP mice 57. Our *in vivo* results demonstrated that TRAMP mice fed a HFD presented increased serum PSA levels, increased TRPV1 expression and increased expression of stem cell markers in prostate tissue, suggesting a more aggressive stage of PCa. TRPV1 levels, along with the levels of the proliferation markers PCNA and Cyclin B1, are significantly reduced in the prostates of mice fed a HFD supplemented with CAP. These findings are consistent with those of Altieri et al., who reported that dietary CAP attenuated cell proliferation and the activity of matrix metalloproteinases 2/9 in an *in vivo* model of urothelial carcinogenesis in rats 58. Similarly, Chen et al. reported that intraperitoneal CAP treatment significantly suppressed proliferation; reduced the expression of Ki-67, Bcl-2, and survivin; and increased the expression of Bax and caspase-3 in the breast tumors of mice 59.

Our findings indicate that TRPV1 is associated with prostate tumor cell growth and proliferation in both *in vitro* and *in vivo* models, and that reducing its expression, either by genetic silencing or capsaicin treatment, exerts an inhibitory effect on cell growth. These observations suggest that TRPV1 may represent a potential therapeutic target, as decreasing its expression in cancers in which it is upregulated could contribute to the control of tumor growth. In addition, the correlation observed between TRPV1 and markers of stemness and resistance may have diagnostic implications in prostate cancer, as tumors exhibiting high TRPV1 expression may be less differentiated, display greater growth potential, and exhibit a more aggressive phenotype.

## Conclusions

Our results demonstrate that TRPV1 is overexpressed in prostate cancer and is associated with tumor proliferation and activation of growth-promoting pathways. TRPV1 expression also correlates with markers of pluripotency and therapy resistance, suggesting a role in maintaining cancer stemness. The antitumor effects of CAP may be mediated, at least in part, by long-term downregulation of TRPV1. These findings support the view that TRPV1 may be regarded as a theragnostic protein, as its expression in prostate tumors could provide prognostic information about the disease, while its downregulation through novel pharmacological approaches could be exploited for therapeutic purposes. However, further studies are warranted to elucidate the cellular mechanisms underlying TRPV1 regulation and the factors influencing its expression changes.

## Supplementary Material

Supplementary figures.

## Figures and Tables

**Table 1:** Antibodies used for Western blot analysis.

Antibodies	Dilution	Reactivity	Reference and source
TRPV1	1:1500	Human	PA5-34498; Thermo Scientific (Waltham, MA, USA)
p21	1:1000	Human	#2947; Cell Signaling Technology (Danvers, MA, USA)
AURKA	1:1000	Human	#14475; Cell Signaling Technology (Danvers, MA, USA)
TRPV1	1:1000	Mouse	ab203103; Abcam (Cambridge, UK)
Cyclin B1	1:1000	Human & Mouse	#4135; Cell Signaling Technology (Danvers, MA, USA)
PCNA	1:1000	Human & Mouse	#13110; Cell Signaling Technology (Danvers, MA, USA)
β-Actin	1:5000	Human & Mouse	A5441; Sigma-Aldrich (St. Louis, MO, USA)
HRP anti-mouse IgG	1:5000	Mouse	A9044; Sigma-Aldrich (St. Louis, MO, USA)
HRP anti-rabbit IgG	1:5000	Rabbit	#7074S; Cell Signaling Technology (Danvers, MA, USA)

The table includes their respective dilutions, references and sources.

**Table 2:** Sequences of the primers used for RT-qPCR analysis of human and mouse samples.

Target	Forward (5'-3')	Reverse (5'-3')
Human-TRPV1	GCCTGGAGCTGTTCAAGTTC	TCTCCTGTGCGATCTTGTTG
Human-Oct4	GACAGGGGGAGGGGAGGAGCTAGG	CTTCCCTCCAACCAGTTGCCCCAAAC
Human-Nanog	TTTGTGGGCCTGAAGAAAC	AGGGCTGTCCTGAATAAGCAG
Human-ABCB1A	TTGCTGCTTACATTCAGGTTTCA	AGCCTATCTCCTGTCGCATTA
Human-Actin	AGAAGGATTCCTATGTGGGCG	CATGTCGTCCCAGTTGGTGAC
Human-18S	GTAACCCGTTGAACCCCATT	CCATCCAATCGGTAGTAGCG
Mouse-TRPV1	CAAGGCTCTATGATCGCAGG	GAGCAATGGTGTCGTTCTGC
Mouse-Oct4	CGGAAGAGAAAGCGAACTAGC	ATTGGCGATGTGAGTGATCTG
Mouse-Nanog	CACAGTTTGCCTAGTTCTGAGG	GCAAGAATAGTTCTCGGGATGAA
Mouse-GAPDH	TGAAGCAGGCATCTGAGGG	CGAAGGTGGAAGAGTGGGAG

**Table 3:**
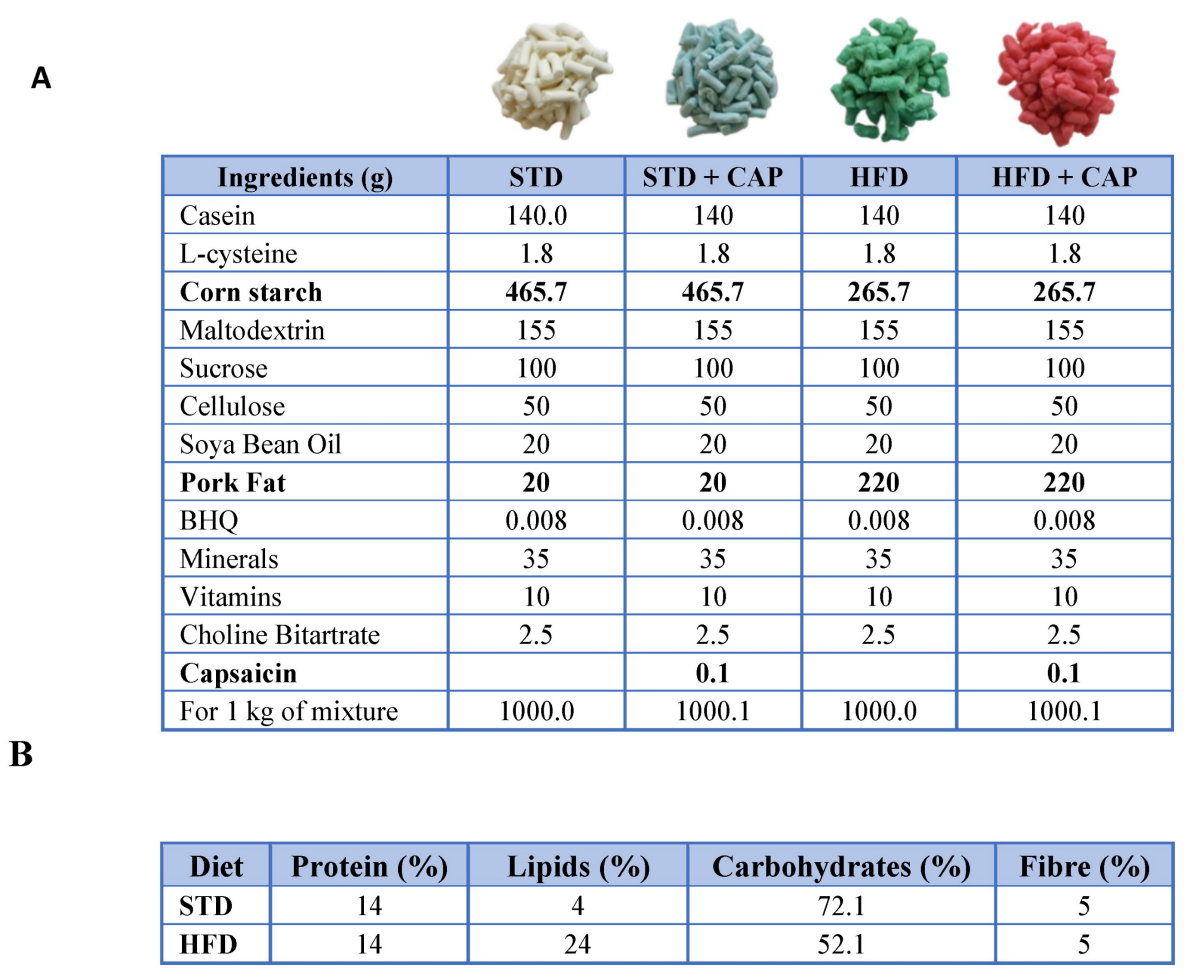
Characteristics of the diets used in the study.

**(A)** Composition of the diets used in the study: STD, STD + CAP, HFD, and HFD + CAP. Ingredients differing between diets are indicated in bold.** (B)** Chemical composition of the feed included casein as the protein source; soybean oil and pork fat as lipid sources; maize starch, maltodextrin, and sucrose as carbohydrate sources; and cellulose as fibre.

**Table 4:** Baseline characteristics of prostate cancer (PCa) patients.

		%	Mean ± SD	Median
**Age** (years)	50-60	12	55.5 ± 0.7	55.5
	60-70	65	65.8 ± 3.0	66.0
	70-80	24	74.0 ± 1.4	74.5
**Weight** (Kg)	60-69	6	64.0 ± 0.0	64.0
	70-79	35	74.2 ± 2.6	74.0
	80-99	41	86.1 ± 5.2	85.0
	> 100	18	107.7 ± 5.9	110.0
**BMI** (Kg/m^2^)	20-25	12	24.3 ± 0.8	24.3
	25-30	53	27.6 ± 1.4	27.2
	> 30	35	33.2 ± 3.2	32.9
**PSA** (ng/mL)	1 - 6.9	41	5.7 ± 1.1	5.9
	7 - 20	29	9.1 ± 2.2	8.1
	> 20	29	25.9 ± 9.6	21.2
**Gleason score**	6	6		
	7	56		
	> 8	38		
**Clinical T stage**	cT1	63		
	cT2	31		
	cT3	6		

The variables "Gleason score" and "Clinical T stage" are categorical; therefore, statistics such as the mean or median do not apply.

## Abbreviations

PCa: prostate cancer
TRPV1: transient receptor potential vanilloid 1
TM: transmembrane
CAP: capsaicin
FBS: fetal bovine serum
SDS-PAGE: SDS-polyacrylamide gel electrophoresis
PEI: Polyethylenimine
TFA: trifluoroacetic acid
TCEP: tris-2(-carboxyethyl)-phosphine
CAA: chloroacetamide
DIA: Data-Independent Acquisition
PASEF: Parallel Accumulation Serial Fragmentation
FDR: False discovery rate
DEPs: differentially expressed proteins
GO: Gene Ontology
KEGG: Kyoto Encyclopedia of Genes and Genomes
TRAMP: Transgenic adenocarcinoma of the mouse prostate
STD: standard diet
STD + CAP: standard diet supplemented with 0.01% w/w capsaicin
HFD: high-fat diet
HFD + CAP: high-fat diet supplemented with 0.01% w/w capsaicin
PSA: prostate-specific antigen
ADT: androgen deprivation therapy
ER: endoplasmic reticulum
PCA: principal component analysis
PCNA: proliferating cell nuclear antigen
ANLN: anillin
AURKA: aurora kinase A
BMI: body mass index
