# Supplementary material for: TRPV1 Downregulation Impairs Prostate Cancer Growth: Functional and Translational Insights from Cellular and In Vivo Models
Source: Int J Biol Sci. 2026 Jan 22;22(4):2101–20. doi: 10.7150/ijbs.125429 (PMC12905635; doi:10.7150/ijbs.125429)
Supplement: Supplementary file 1 — Supplementary figures. [file ijbsv22p2101s1.pdf]

SUPPLEMENTARY FIGURES

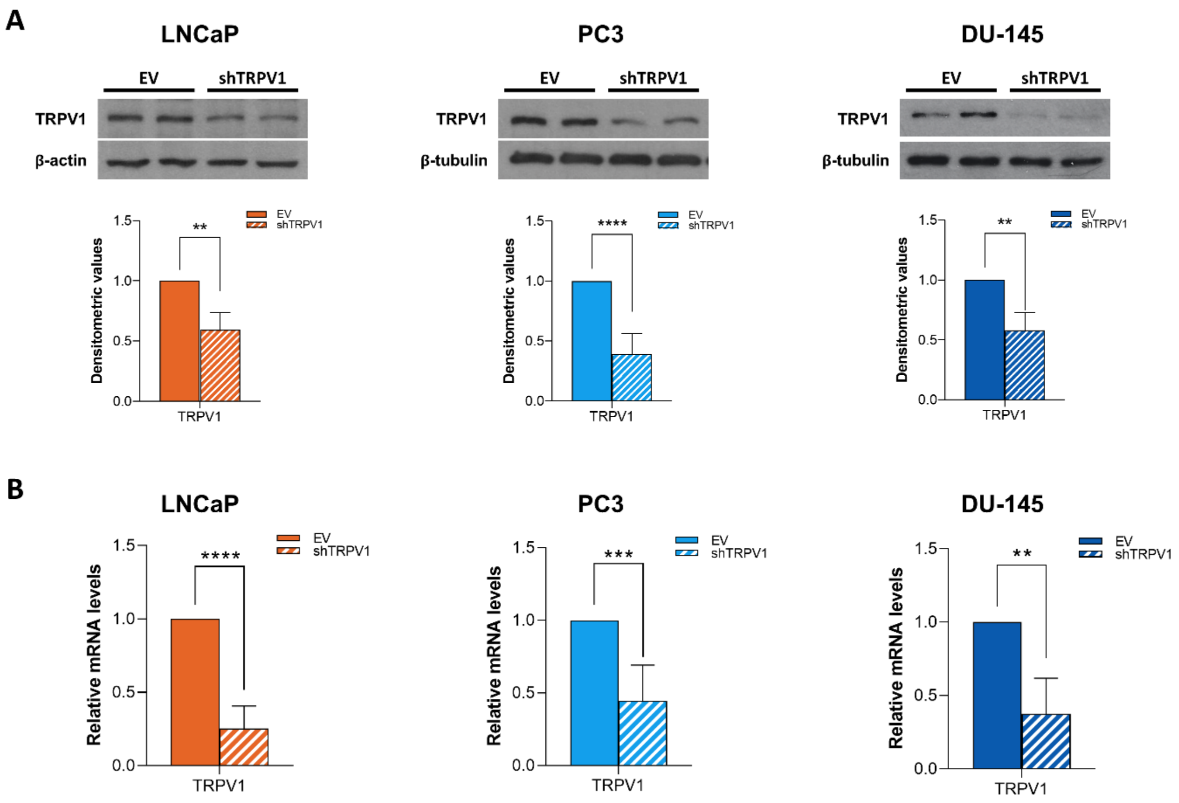

Supplementary Figure 1. Knockdown efficiency of TRPV1 in different cancer cell lines. LNCaP, PC3, and DU-145 cells were transduced with either an empty vector (EV) or an shTRPV1 vector via lentivirus-mediated infection. **(A)** TRPV1 protein expression levels were determined by Western blot analysis, with β-actin or β-tubulin serving as loading controls. Densitometric analyses of the bands represent the mean ± SD from five independent experiments. **(B)** TRPV1 expression levels were determined by RT-qPCR. Data represent relative expression normalized to actin, used as the housekeeping gene. Results are presented as mean ± SD of six independent experiments. Statistical significance was determined using a paired t-test: $p < 0.05$ (*), $p < 0.01$ (**), $p < 0.001$ (***) and $p < 0.0001$ (****).

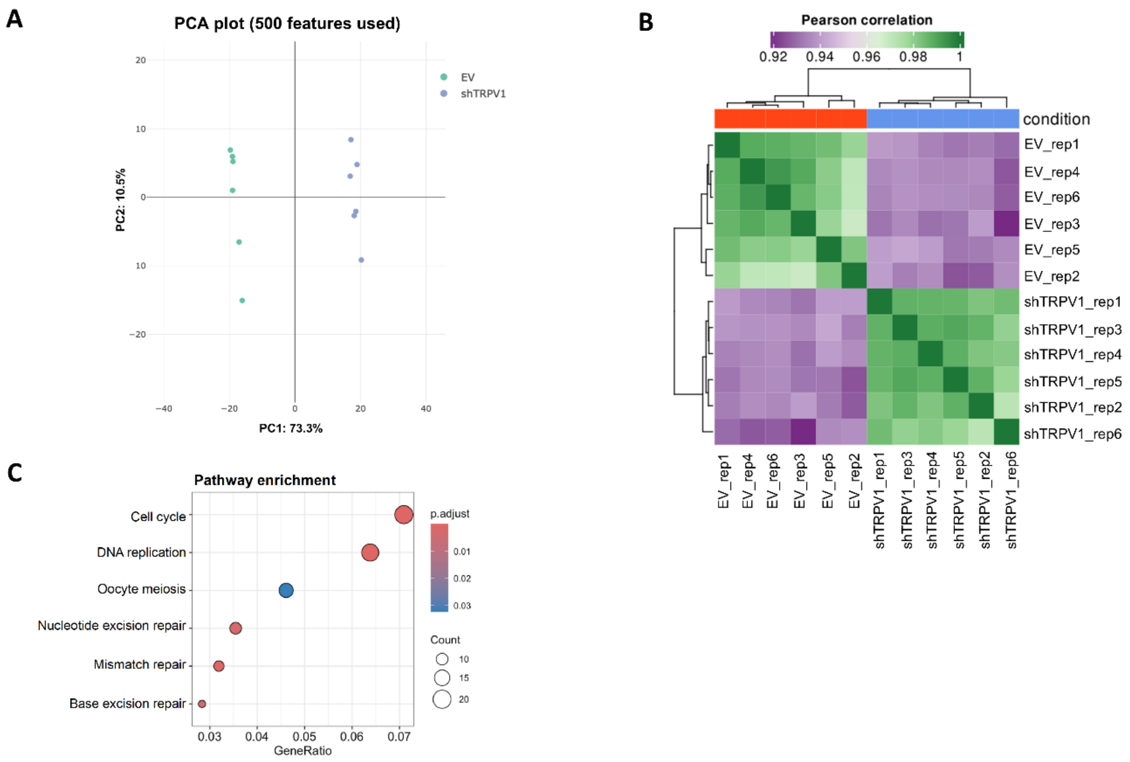

Supplementary Figure 2. Complementary analyses of the proteomic assay comparing EV- and shTRPV1-infected PC3 cells. (A) Principal component analysis (PCA) based on the 500 most variable features across all samples. PC1 distinguishes between conditions “empty vector” (green) and “shTRPV1 vector” (blue), whereas PC2 accounts for differences among biological replicates. **(B)** Sample correlation matrix illustrating Pearson correlation coefficients between EV and shTRPV1 samples. Correlations ≥ 0.96 are represented in green, whereas those < 0.96 are shown in purple. **(C)** KEGG (Kyoto Encyclopedia of Genes and Genomes) pathway enrichment analysis of the differentially expressed proteins (log2-fold change $> |1|$, $p < 0.05$. Panels (A) and (B) were generated via the FragPipe-Analyst platform, whereas panel (C) was generated via in the R programming environment.

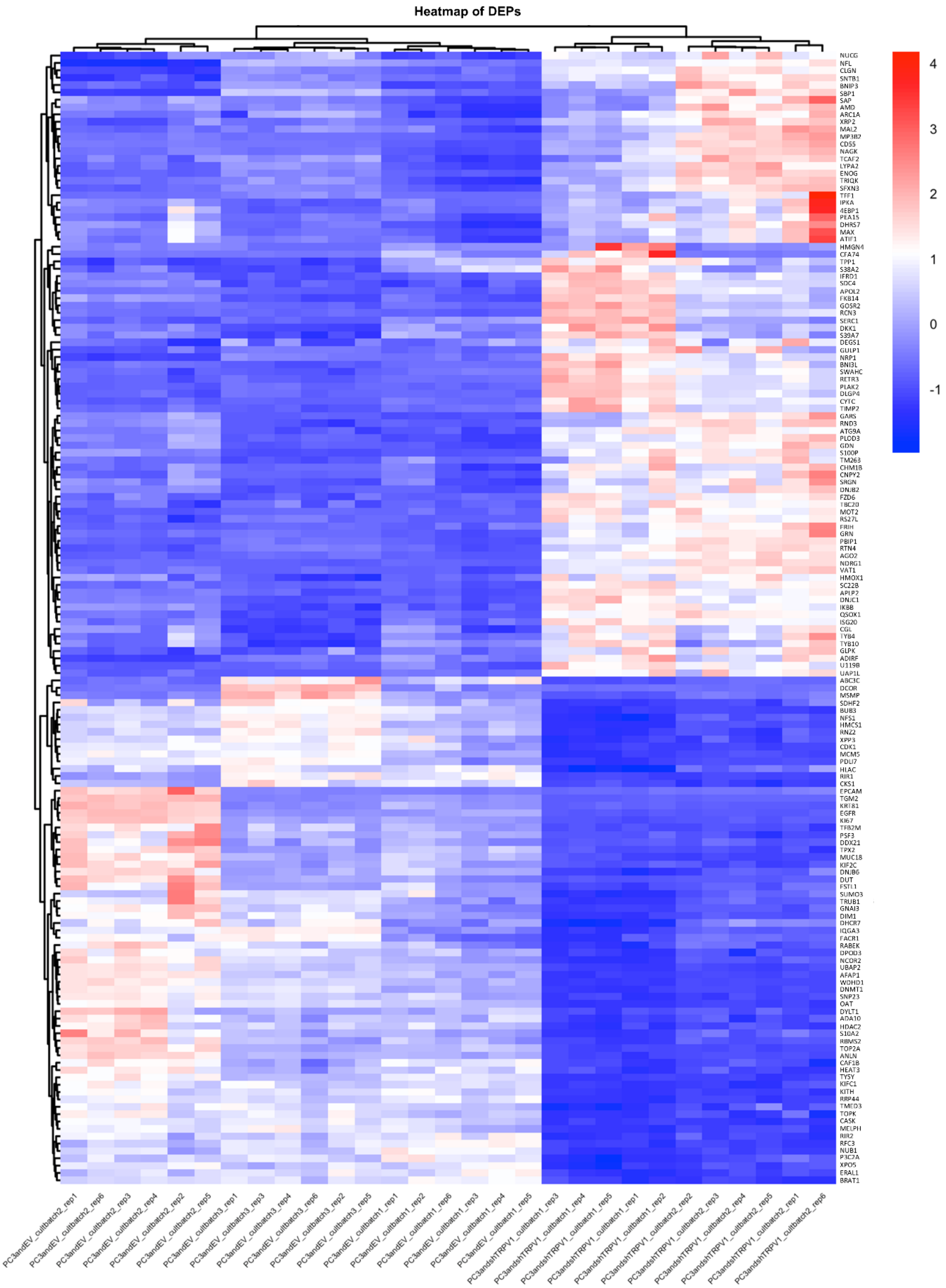

Supplementary Figure 3. Enlarged view of the heatmap from Figure 2B. Heatmap of differentially expressed proteins (DEPs) (log2-fold change > |1.2|) between cells infected with the EV or shTRPV1 vector. The heatmap displays hierarchical clustering of both samples and proteins.

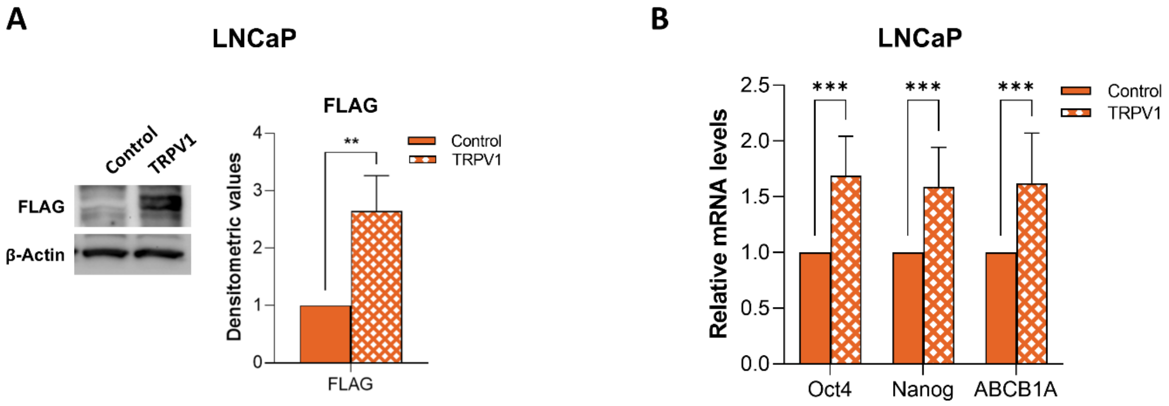

Supplementary Figure 4. Effect of TRPV1 overexpression on stemness-related markers. TRPV1 was overexpressed in LNCaP cells by transfection with a FLAG-tagged plasmid (TRPV1_OHu19934D_pcDNA3.1+/C-(K)-DYK). (A) Detection of the FLAG tag in TRPV1-overexpressing LNCaP cells by Western blotting. β-Actin served as a loading control. Densitometric analyses represent the mean ± SD of three independent experiments. (B) mRNA expression of the stemness markers Oct4, Nanog, and ABCB1A. Transcript levels were quantified by RT-qPCR, normalized to actin as the housekeeping gene, and are presented as mean ± SD of three independent experiments. $p < 0.05$ (*), $p < 0.01$ (**), $p < 0.001$ (***), and $p < 0.0001$ (****) indicate significant differences according to two-way ANOVA with Sidak's multiple comparisons test.

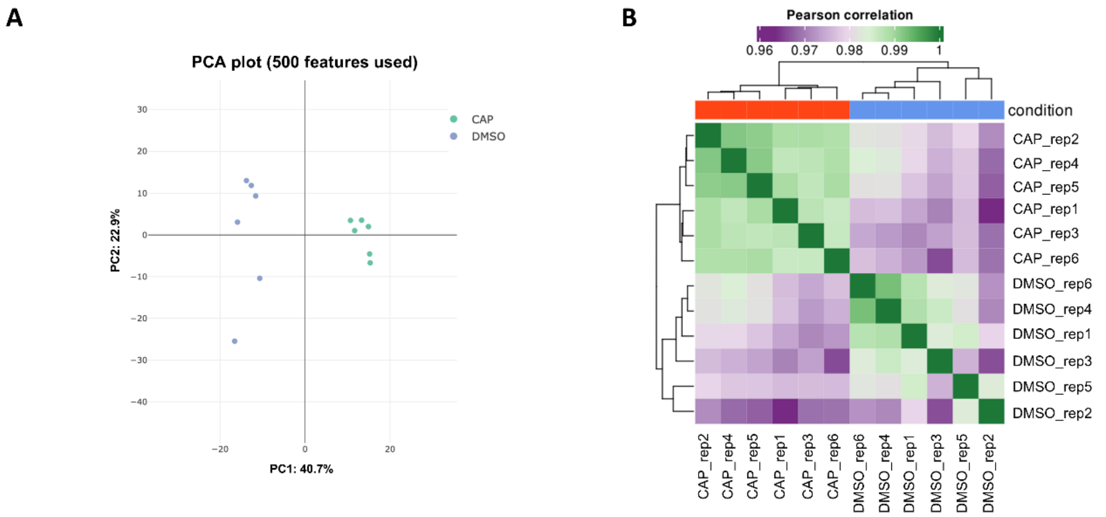

Supplementary Figure 5. Complementary analyses of the proteomic assay comparing DMSO- and CAP-treated PC3 cells. (A) Principal component analysis (PCA) plot showing the variance in protein expression profiles based on the 500 most variable features across all samples. PC1 distinguishes between conditions, treated with “CAP” (green) or with “DMSO” (blue), whereas PC2 accounts for differences among biological replicates. **(B)** Sample correlation matrix illustrating the Pearson correlation coefficients between DMSO- and CAP-treated samples. Correlations ≥ 0.98 are represented in green, whereas those < 0.98 are shown in purple. Graphs were generated via the FragPipe-Analyst platform.
